# β-methylamino-L-alanine (BMAA) is not found in the brains of patients with confirmed Alzheimer’s disease

**DOI:** 10.1038/srep36363

**Published:** 2016-11-08

**Authors:** Julie P. Meneely, Olivier P. Chevallier, Stewart Graham, Brett Greer, Brian D. Green, Christopher T. Elliott

**Affiliations:** 1 Institute for Global Food Security, School of Biological Sciences, Queens University Belfast, BT7 1NN, Northern Ireland; 2 Beaumont Research Institute, 3811 W. 13 Mile Road, Royal Oak, Michigan 48073, USA

## Abstract

Controversy surrounds the proposed hypothesis that exposure to β-methylamino-L-alanine (BMAA) could play a role in various neurodegenerative conditions including Alzheimer’s disease (AD). Here we present the results of the most comprehensive scientific study on BMAA detection ever undertaken on brain samples from patients pathologically confirmed to have suffered from AD, and those from healthy volunteers. Following the full validation of a highly accurate and sensitive mass spectrometric method, no trace of BMAA was detected in the diseased brain or in the control specimens. This contradicts the findings of other reports and calls into question the significance of this compound in neurodegenerative disease. We have attempted to explain the potential causes of misidentification of BMAA in these studies.

β-methylamino-L-alanine (BMAA) is a naturally occurring, non-essential, non-proteinogenic amino acid. It has been hypothesised to play a causal role in various neurodegenerative diseases such as amyotrophic lateral sclerosis/Parkinsonism dementia complex (ALS/PDC) in the Chamorro people living in the South Pacific Island of Guam^1^,^2^,^3^,^4^ and Alzheimer’s disease (AD) in a small cohort of Caucasian North Americans^3^,^4^. Epidemiological studies on Guam were undertaken from 1945 until 1983 looking at the high incidences of ALS and PDC and it was concluded that the underlying causes of these neurological diseases were probably due to environmental factors related to the Chamorro traditions, although no specific factors were identified at that stage^5^,^6^. No pre-existing medical conditions, significant differences in lifestyle or exposure were observed^5^,^6^. The epidemiology was not consistent with Mendelian inheritance^7^, however all patients tended to be from lower social backgrounds, be less educated, eat more traditional foods (home grown foods, raw meat and fish) and have more contact with animals; thus dietary elements were implicated in the aetiology of the diseases^5^,^6^. In 1967, Vega & Bell isolated BMAA from cycad seeds that were used to prepare flour for tortillas, dumplings and soup by the Chamorro people^8^. They investigated its effects in chicks and rats and observed weakness, convulsions and loss of coordination and thus it was proposed that this compound could be the neurotoxin toxin responsible for ALS/PDC^8^. Further animal studies demonstrated that primates orally dosed with 100 to 250 mg/kg L-BMAA for up to 12 weeks showed signs of neuropathological changes consistent with ALS/PDC, however the animals received relatively large doses of BMAA and motor dysfunction onset was rapid unlike the time lapse exhibited for ALS/PDC in Guam which was over decades^9^. On that basis the authors expressed substantial reservations about linking these observations to ALS/PDC.

Analysis of BMAA concentrations in cycad seeds by various research groups have highlighted that the toxin was present in the seed gametophyte tissue at concentrations of between 290 to 1560 μg/g (dry weight)^10^, 240 μg/g (dry weight)^11^ and 593 μg/g (dry weight)^12^. Subsequent investigations by Duncan *et al*.^13^ demonstrated that at least 85% of BMAA was removed from cycad flour during processing and it was improbable that consumption of such low concentrations of the toxin would cause the neurological symptoms associated with ALS/PDC. In a pharmacokinetic study conducted in rats by this research group, they observed BMAA at concentrations of >250 μM in the brain following doses of >100 mg/kg. This implied that the BMAA dose through dietary consumption of cycad seeds alone was several orders of magnitude lower than that required to replicate the previously reported neurotoxicity in the rat model^14^. Another proposed dietary route and the suggestion of bio-magnification was offered by Cox and Sacks^15^. The consumption of Guamanian flying foxes which fed on the cycads was core to the Chamorro tradition. The decline of the flying fox population correlated with the drop in the number of cases of ALS/PDC presented in Guam which matched the epidemiology of these diseases. Banack and Cox analysed the skin of three preserved museum flying fox specimens (collected 5 decades previously) and found BMAA concentrations to be between 1287 to 7502 μg/g. They proposed that “Traditional feasting on flying foxes may be related to the prevalence on neuropathologic disease in Guam”^16^. The source of BMAA in cycad seeds was investigated by Cox and his colleagues^3^. They found BMAA (0.3 μg/g) was produced by *Nostoc* cyanobacteria, root symbionts of the cycad trees and reported that bio magnification of BMAA through the food chain of the indigenous population of Guam may explain the high incidences of neurodegenerative disease. On further analysis, the researchers reported that BMAA was present in both free and bound form and after acid hydrolysis of protein fractions of the cyanobacteria, 72 μg/g of bound BMAA was quantified (240X the recorded free level)^2^. The implication that the neurotoxin was produced by cyanobacteria had huge ramifications for public health worldwide as cyanobacteria are ubiquitous not only in freshwater systems but colonise a wide variety of habitats^17^,^18^,^19^. In 2005, Cox *et al*.^20^ described the presence of BMAA in a wide variety of cyanobacterial genera and strains from all over the globe, with concentrations of up to 6478 μg/g for free BMAA and 5415 μg/g for protein bound BMAA^20^. Other researchers have investigated the presence of BMAA in cyanobacterial extracts and substantiated the above findings^21^,^22^ while some have observed much lower concentrations^23^,^24^ and several research groups have found no evidence of BMAA production by cyanobacteria by other methods^12^,^25^,^26^. The idea of cyanobacterial production of BMAA led to investigations in higher trophic organisms and low concentrations were reported in zooplankton, mussels, oysters and various species of fish^22^,^24^,^27^,^28^, thus indicating that the global human population could be exposed to this compound through the food web. With cyanobacteria being linked to the production of BMAA, the implications for drinking water were highlighted^21^ thus driving the research further.

The findings of free BMAA in brain tissue from 6 Chamorro patients who had suffered from ALS/PDC and 2 Canadian AD patients coupled with the fact that no BMAA was found in tissue from 13 Canadian individuals who had died from unrelated causes^3^ fuelled the hypothesis that BMAA was linked to the aetiology of neurodegenerative diseases. These findings were profound in terms of the possible link between this non-proteinogenic amino acid and its potential causal role not only in ALS/PDC exhibited by a small cohort on the island of Guam but also for the debilitating, neurological degenerative condition of AD. These researchers, subsequently reported both the free and bound forms of BMAA in the brains of 8 Chamorro ALS/PDC patients, 2 Canadian AD patients^1^ and in North American ALS and AD patients 4. Contrary to this, Montine *et al*.^29^ detected no free BMAA in the brains of control subjects, patients with AD or Chamorro people with and without PDC. Despite further improvement of methodologies^30^,^31^, this group were still unable to replicate the results of Cox *et al*.^3^. A further investigation by Combes *et al*.^32^ also failed to detect BMAA in brain samples from patients (n = 2) who died of ALS. More recently Karlsson *et al*.^33^ demonstrated a dose-dependent increase in free and bound BMAA in tissue of rat neonates, with levels in the liver being much higher than concentrations in the brain. BMAA was found to clear over time as samples from adult rats showed no detectable concentrations of either free or bound BMAA. This contradicts the earlier hypothesis that BMAA could accumulate over time and may lead to the onset of ALS, PDC or AD^2^ but is in agreement with a pharmacokinetic study performed in rats^14^.

The purpose of this study was to develop and fully validate a powerful method for the determination of free and bound BMAA in brain tissue using dansyl chloride derivatization^34^,^35^ and apply the method to the largest sample set of brains from AD patients (n = 20) and controls (n = 20) to date.

## Results

**Figure 1:**
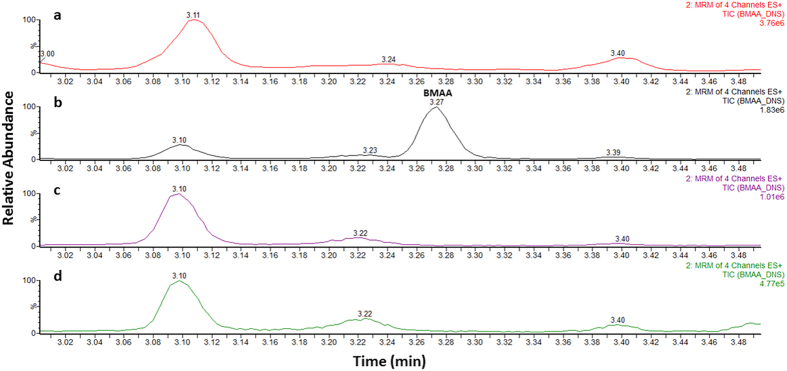
Ultra-high performance liquid chromatography-tandem mass spectrometry analysis of free BMAA extracts. Chromatograms of (**a**) blank lamb’s brain extract, (**b**) a matrix matched standard solution of BMAA, (**c**) control patient’s brain extract and (**d**) alzheimer’s diseased brain extract. (MRM, multiple reaction monitoring).

**Figure 2:**
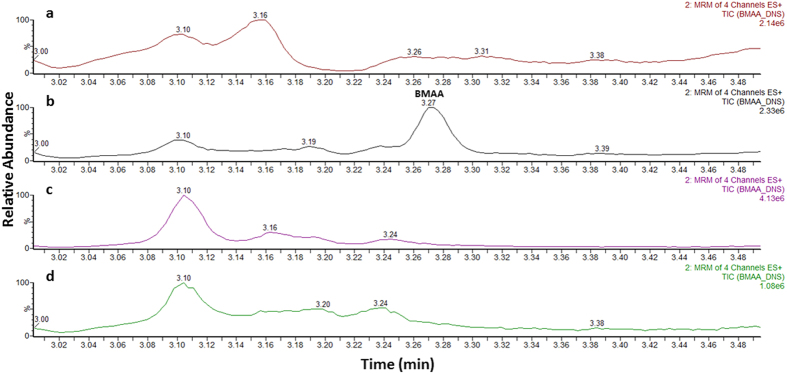
Ultra-high performance liquid chromatography-tandem mass spectrometry analysis of protein-bound BMAA extracts. Chromatograms of (**a**) blank lamb’s brain extract, (**b**) a matrix matched standard solution of BMAA, (**c**) control patient’s brain extract and (**d**) alzheimer’s diseased brain extract. (MRM, multiple reaction monitoring).

A seven-point calibration curve was constructed between 0.2 μg/g and 10 μg/g of BMAA for the determination of the concentrations of free BMAA in the samples and between 2 μg/g and 40 μg/g for protein-bound BMAA. The chromatograms for free (blank and 2 μg/g) and bound (blank and 8 μg/g) lamb’s brain matrix are displayed in Figs 1a,b and 2a,b, respectively. Linear regression analysis with 1/x weighting for the curve fitting was used to evaluate this parameter. The linearity was assessed over three days; for both free and bound BMAA calibrations curves r^2^ > 0.99. All calibrators for all three days fell within ±10% of the expected concentration value.

The precision of the method was determined by calculating the intra-day (within run) and inter-day (between run) variation expressed as the relative standard deviation (% RSD) for both the free BMAA (0.5 μg/g, 3 μg/g, 9 μg/g) (Table 1) and protein-bound BMAA (3 μg/g, 9 μg/g, 30 μg/g) (Table 2).

Decision limits (ccα) for both free and protein-bound BMAA were calculated using fortified blank samples. The signal was plotted against the added concentration and the corresponding concentration at the *y*-intercept plus 2.33X the standard deviation of the within-laboratory reproducibility of the intercept was determined as the decision limit. The resulting decision limits were determined as 0.4 μg/g and 0.7 μg/g for free and protein-bound BMAA, respectively. Detection capabilities (ccβ) for both were calculated by taking the concentration at the decision limit plus 1.64X the standard deviation of the within-laboratory reproducibility of the mean measured content at the lowest concentration of the spiked blank. The results indicated detection capabilities of 0.7 μg/g and 1.2 μg/g for free and protein-bound BMAA, respectively.

Recovery was verified by experiments using fortified blank material as described for the precision of the methods. The mean recovery for free BMAA was calculated as 101%, 104% and 99% for concentrations at 0.5 μg/g, 3 μg/g and 9 μg/g, respectively. For protein-bound BMAA the mean recovery was found to be 97%, 101% and 101% at 3 μg/g, 9 μg/g and 30 μg/g, respectively. Absolute recovery was also determined for free BMAA and protein-bound BMAA by comparing results of samples spiked prior to extraction and those spiked after extraction but before derivatization. For free BMAA, the brain sample was spiked at 2 μg/g while for protein-bound BMAA, the brain sample was fortified at 4 μg/g and the results are shown in Tables 1 and 2.

**Figure 3:**
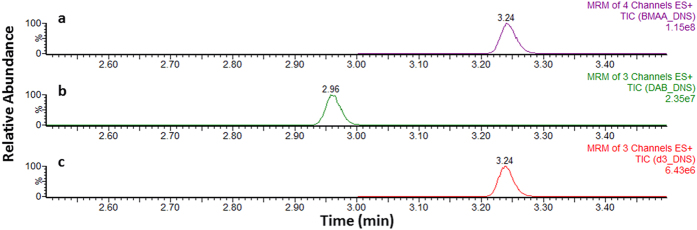
Ultra-high performance liquid chromatography-tandem mass spectrometry chromatograms obtained for (**a**) BMAA-DNS, (**b**) DAB-DNS and (**c**) D_3_-BMAA-DNS solvent standards at 250 ng/ml, 250 ng/ml and 100 ng/ml, respectively. (MRM, multiple reaction monitoring).

Blank samples (n = 20) were extracted as per the methods and analysed with the calibrators. The results displayed no co-eluting peak with the 5-dimethylamino-1-naphthalenesulfonyl chloride/dansyl chloride (DNS) derivatized BMAA (BMAA-DNS) at 3.24 minutes. In addition, chromatographic separation of dansyl chloride derivatized L-2,4-diaminobutyric acid (DAB-DNS) was achieved, showing a retention time of 2.96 minutes (Fig. 3). The other isomers of BMAA, namely β-Amino-*N*-methyl-alanine (BAMA) and *N*-(2-aminoethyl) glycine (AEG) were not available to us for testing at this time.

The validated quantitative method was applied to the brain tissue from AD patients (n = 20) and from healthy age-matched subjects who had not suffered from the disease (n = 20). Samples were extracted according to the protocols described and deuterium labelled BMAA (D_3_-BMAA) internal standard was used for quantification. A blank sample was extracted and used as negative control throughout the analysis to determine if there was any carry-over in the system. BMAA (free or protein-bound) was not detected in any of the control samples or the diseased samples tested at or above the decision limit of the method (0.7 μg/g and 1.2 μg/g for free and protein-bound BMAA, respectively) (Figs 1c,d and 2c,d).

## Discussion

Controversy surrounds the proposed hypothesis that BMAA exposure through consumption of contaminated food such as fish, shellfish^22^,^24^,^27^,^28^,^35^ and drinking water via cyanobacteria^20^,^21^,^22^ could play a causal role in various neurodegenerative pathological conditions including AD^2^,^3^,^4^. Here we present the results of our study in which we analysed brain samples from patients with pathologically confirmed AD, (n = 20), and from subjects who did not suffer from this neurodegenerative disease; control subjects (n = 20). To the authors’ knowledge this is the largest investigation confirming the presence/absence of BMAA in the brains of patients’ who suffered from AD. No BMAA, either free or protein-bound was detected in the diseased brains or in the control samples. This is in agreement with other researchers^29^,^30^,^31^, using different methodologies, however, these findings contradict the observations of another group of researchers^2^,^3^,^4^ who claim the total BMAA concentrations (free and protein-bound) found in the brains of those who suffered from AD ranged from 7 μg/g to 236 μg/g.

The method developed was rigorously validated as a quantitative procedure for the determination of free and protein-bound BMAA in brain using EC/657/2002 as guidance^36^. Intra- and inter-day precision, expressed as the relative standard deviation (RSD%) for both free and protein-bound BMAA showed results <9% (Tables 1 and 2) and are within the acceptance criteria of the guidelines. Acceptable linearity (r^2^ > 0.99) was also displayed for the matrix matched calibrations curves for free and bound BMAA with the calibrators all falling within ±10% of the expected value. Observed mean recoveries of the samples spiked at three concentrations for both free and protein-bound BMAA ranged from 97% to 104% which again fall within the tolerances outlined in the guidelines as did the absolute recoveries which were calculated as 80–105%. The decision limits (limits of detection) of 0.4 μg/g and 0.7 μg/g for free BMAA and protein-bound BMAA and detection capabilities (limits of quantification) of 0.7 μg/g and 1.2 μg/g for free BMAA and protein-bound BMAA meet the sensitivity requirements based on the reported concentrations of BMAA found in diseased brains as outlined above^2^,^3^,^4^. Importantly, this method ensured the separation of the commonly found isomer DAB from BMAA and there were no co-eluting peaks with BMAA when twenty blank brain samples were analysed.

The sample preparation was based on established methods published in the scientific literature^37^,^38^. Samples were sonicated in water to extract the free amino acids and acetone added to precipitate out the proteins^33^, the resultant supernatant was dried and reconstituted prior to derivatization. Although this protocol differs from that performed by those who found BMAA present in AD brain^1^,^2^,^3^,^4^, it has been shown to be applicable and successful in extracting free BMAA from rat brain^33^. Through the full validation of the method, it has been demonstrated that it is an efficient method of extraction, showing recoveries of >90%. This concurs with a recent comparative study performed by Lage *et al*.^38^.

The extraction of protein-associated BMAA is obviously more complex and requires the release of BMAA through protein hydrolysis. Prior to applying conventional HCl hydrolysis, the protein pellet of the sample was subjected to further protein precipitation using 10% TCA (2 cycles). 6 M HCl was then added and hydrolysis performed at 110˚C overnight (17 hours)^33^. Following SPE clean-up^23^, the eluate was dried and reconstituted prior to derivatization. Again to ensure the efficiency of this sample preparation, brain samples were spiked before and after extraction and the recoveries determined as >80%. An internal standard (D_3_-BMAA) had also been incorporated for control throughout the process and was shown to be stable during the entire procedure. This was congruent with previous reported findings^12^,^23^,^35^.

The final stage of sample preparation involved derivatization of the sample prior to ultra-high performance liquid chromatography-tandem mass spectrometry (UPLC-MS/MS). One of the most commonly used methods is 6-aminoquinolyl-N-hydroxysuccinimidyl carbamate (AQC) derivatization, however our approach was the use of DNS that had been successfully presented for BMAA analysis by Salomonsson *et al*.^35^. The researchers had compared the sensitivity of both methods in mussel samples and concluded that DNS was a good alternative. Questions have arisen regarding the use of AQC due to the huge discrepancies in data reported by different research groups. In one study, concentrations of BMAA in cyanobacteria were reported to be present at concentrations ranging from 8 μg/g to 287 μg/g^21^, while in another the concentrations ranged from 0.001 μg/g to 0.015 μg/g^24^.

As reported by Faassen^39^, such variability in concentration can be explained by the overall quality of the analytical method used to quantify BMAA. The analytical method used in initial report of BMAA in human brain samples for instance can be considered outdated and replaced by more sensitive, selective and well described procedures^32^. Indeed, as outlined by Faassen, only 16 of the 44 studies presented in the literature were able to provide enough evidence for correct BMAA analysis^39^. It is the opinion of the authors of the present study that inadequately robust analytical methods in all likelihood combined with poor data interpretation and reporting are responsible for the incorrect identification of BMAA and this has fuelled the controversies surrounding BMAA.

By the application of the most stringent validation criteria and the methodology developed by an expert mass spectrometry group, the subsequent data reported can be considered as fit for purpose. Thus the failure to detect any BMAA presence in brain tissue from AD patients is a scientifically robust finding.

In this study a highly sensitive and selective method was developed and fully validated for the detection and quantification of free and protein-bound BMAA in post-mortem human brain tissue. The method was shown to quantify derivatized BMAA at comparative concentrations reported by other researchers investigating the presence/absence of this putative neurotoxin in the brains of patients with AD. The results of our study seriously contest previous findings that BMAA accumulates in the brains of those suffering from Alzheimer’s disease^2^,^3^,^4^ and therefore seriously question whether this compound plays any role in the disease whatsoever.

## Materials and Methods

### Chemicals

BMAA hydrochloride, L-2,4-diaminobutyric acid (DAB) dihydrochloride, trichloroacetic acid (TCA), 5-dimethylamino-1-naphthalenesulfonyl chloride/dansyl chloride (DNS), formic acid, acetonitrile, methanol, hydrochloric acid (HCl) [37%], chloroform and Corning^®^ Costar^®^ Spin-X^©^ centrifuge filters were purchased from Sigma-Aldrich Company Ltd (Gillingham, United Kingdom). All chemicals used were of analytical grade or higher. The water was purified using a Milli-Q purification system from Merck Millipore (Merck KGaA, Darmstadt, Germany). Deuterium labelled BMAA (D_3_-BMAA) was a gift from Johan Rosén at The National Food Agency in Sweden, and was synthesized as described in the publication by Rosén and Hellenäs^12^. Stock solutions of BMAA (1 mg/ml) were prepared in Milli-Q water and stored at −20˚C.

### Equipment

Mass spectrometry was performed using an ACQUITY UPLC I-Class coupled to a Xevo TQ-S (triple quadrupole MS/MS) mass spectrometer (Waters, Manchester, UK) equipped with an Acquity UPLC BEH C_18_ column (1.7 µm, 2.1 × 50 mm) (Waters, Wexford, Ireland).

### Samples

Frozen human post-mortem (PM) brain tissue was kindly provided by The South West Dementia Brain Bank (SWDBB) laboratory, Southmead Hospital (Bristol, UK) and the Newcastle Brain Tissue Resource, both licensed by the Human Tissue Authority (UK). All subjects donated their brains in strict accordance with ethically approved procedures. Control samples (n = 20) and samples from pathologically confirmed cases of AD (n = 20) were taken from the neocortex, Brodmann 7 region of the brain, known to exhibit AD pathology^40^. All confirmed AD samples were selected by neuropathologic assessment according to a harmonised protocol adopted by the Brains for Dementia Research Neuropathology group^41^. Details of the samples are included in Table 3. Fresh, frozen, lamb’s brain, free from preservatives, was purchased from Samples for Schools (Hampshire, UK) to enable method development and validation prior to testing of the human brain tissue.

### Sample preparation

The frozen tissue samples were lyophilised and milled under liquid nitrogen (SPEX CertiPrep Freezer/Mill). Sample preparation was performed according to Karlsson *et al*.^33^ with some modifications. 25 mg of lyophilised tissue was spiked with 25 μL of the deuterated BMAA internal standard, (D_3_-BMAA, 4 μg/mL). 400 μL Milli-Q water was added and the sample sonicated for 15 minutes in an ice water bath. The homogenate was mixed with 1.6 mL of ice-cold acetone, and kept on ice for 30 minutes to allow the protein to precipitate. Subsequently the samples were centrifuged for 5 minutes at 4,500× *g* and the protein pellet separated from the supernatant. The supernatant was evaporated under nitrogen at 80 °C and the residue derivatized (see below) to determine free BMAA in the samples.

For the determination of protein-bound BMAA, the protein pellet was rinsed with 200 μL of cold acetone to remove any residual free BMAA and protein precipitation was performed with 1.8 ml 10% TCA (30 minutes on ice) for two cycles. The sample was spiked with 25 μL of the internal standard (D_3_-BMAA, 4 μg/mL). Release of protein-bound BMAA was achieved by the addition of 600 μL 6 M hydrochloric acid to the pellet and incubation at 110 °C overnight (17 hours). The hydrolysate was centrifuged at 16,100× *g* (Costar Spin-X^©^ centrifuge filters) for 5 minutes and dried under nitrogen at 80 °C. Sample clean-up was based on the method described by Jiang *et al*.^23^. The residue was reconstituted in 550 μL of Milli-Q water and liquid-liquid extraction performed using 1 mL of chloroform. The sample was mixed for 5 minutes on a roller mixer and centrifuged at 4,500× *g* for 3 minutes. 500 μL of the aqueous layer was transferred to a new eppendorf tube and 500 μL of 0.2% (v/v) formic acid in water added prior to solid phase extraction (SPE).

SPE was performed using Isolute HCX columns (Isolute HCX-3, 100 mg, Sorbent AB, Sweden). The column was conditioned with 1 mL of methanol and equilibrated using 1 mL of 0.1% (v/v) formic acid solution. 1 mL of sample solution was loaded, and the column washed with 1 mL of 0.1% (v/v) formic acid solution followed by 1 mL of methanol. Elution of BMAA was achieved using 2 × 800 μL fractions of saturated ammonium hydroxide solution (NH_4_OH) and methanol (5:95, v/v). The eluate was dried under nitrogen at 50 °C, and derivatized.

The method used for derivatization was adapted from that first described by Zhang *et al*.^34^ and used for BMAA by Salomonsson *et al*.^35^. Briefly, the dry residue was reconstituted in 250 μL of 0.2 M borate buffer (pH 9.5) and 250 μL dansyl chloride (DNS) (10 mg/mL in acetone) added. The tube was vortexed and placed in a water bath at 60 °C for 1 hour. The derivatized sample was filtered using a centrifugal filter (Costar Spin-X^©)^ for 30 seconds, and 300 μL transferred into a HPLC vial. Finally, 300 μL of methanol was added and 1 μL of the mixture was injected onto the LC/MS instrument.

### Ultra-performance liquid chromatography tandem mass spectrometry [UPLC-MS/MS]

Analysis of the brain samples was performed using an Acquity UPLC I-Class system coupled to a Xevo TQ-S (triple quadrupole MS/MS) mass spectrometer (Waters, Ireland). The derivatized analytes were separated on an Aquity UPLC BEH C_18_ column (1.7 μm, 2.1 × 50 mm) (Waters, Wexford, Ireland) maintained at 60 °C in a column oven. The mobile phase consisted of (A) 0.1% formic acid in water and (B) 0.1% formic acid in methanol and was delivered as a gradient at a flow rate of 600 μL/min. The gradient was as follows: 0.0 min, 35% B; 0.5 min, 35% B; 4.0 min, 75% B; 5.0 min, 99% B; 6.25 min, 99% B; 6.3 min, 35% B and 7.0 min, 35% B.

The instrument was operated in electrospray positive mode and the ionization parameters were as follows: capillary voltage 1.5 kV, cone voltage 20 V, source offset 60 (Arbitrary unit), source temperature 150 °C, desolvation temperature 450 °C and desolvation gas flow 800 L/h. Targeted detection and quantification were achieved using multiple reaction monitoring (MRM) with the transitions optimized manually to achieve maximum sensitivity (Table 4). The samples were evaluated using TargetLynx software (Waters, Ireland).

### Method Validation

Due to the scarcity and precious nature of PM human brain, validation was performed using commercially available lamb’s brain as large numbers of samples are required to complete the appropriate analytical testing. Prior to validation, the lamb’s brain sample was lyophilised, milled under liquid nitrogen and tested to ensure it contained no traces of BMAA. Working standards at concentrations of 40, 20, 10, 8, 6, 4, 2, 1, 0.4 and 0.2 μg/ml were prepared in Milli-Q water to create the matrix matched calibration curves for both free and bound BMAA. Standard solutions for the fortification of blank material were prepared at 30, 9, 3 and 0.5 μg/ml in Milli-Q water.

Calibration curves were constructed by spiking the brain matrix (25 mg) at concentrations of 0.2, 0.4, 1, 2, 4, 8 and 10 μg/g for the detection of free BMAA and at 2, 4, 6, 8, 10, 20 and 40 μg/g for protein-bound BMAA using the working standards described above. To validate the free BMAA method, six replicates were spiked at 0.5 μg/g, six at 3 μg/g and six at 9 μg/g BMAA while for validation of the bound BMAA method six replicates were spiked with 3 μg/g, 9 μg/g and 30 μg/g of BMAA to cover the range of each calibration curve. D_3_-BMAA was incorporated as described previously (Sample Preparation). All calibrators/samples were extracted as outlined earlier and the validation performed over three consecutive days. The parameters assessed were linearity, specificity, intra- and inter-day precision, recovery, absolute recovery, the decision limit (ccα) (limit of detection, LOD) and the detection capability (ccβ) (limit of quantification, LOQ).

## Additional Information

**How to cite this article**: Meneely, J. P. *et al*. β-methylamino-L-alanine (BMAA) is not found in the brains of patients with confirmed Alzheimer’s disease. *Sci. Rep.* **6**, 36363; doi: 10.1038/srep36363 (2016).

**Publisher’s note:** Springer Nature remains neutral with regard to jurisdictional claims in published maps and institutional affiliations.

## Figures and Tables

**Table 1:** Validation data for free BMAA.

	Fortified Lamb’s Brain Samples		
	0.5 μg/g	3 μg/g	9 μg/g
Intra-day RSD (%) (n = 6)	8.4	4.7	1.7
Inter-day RSD (%) (n = 18)	7.7	4.7	1.9
Average conc. (repeatability) (μg/g)	0.48	3.00	8.92
Average conc. (reproducibility) (μg/g)	0.51	3.13	8.93
Absolute recovery (%)*	90	105	95

^*^Absolute recovery expressed as the extraction efficiency when spiked at 2 μg toxin per g brain.

**Table 2:** Validation data for protein-bound BMAA.

	Fortified Lamb’s Brain Samples		
	3 μg/g	9 μg/g	30 μg/g
Intra-day RSD (%) (n = 6)	6.5	2	4.6
Inter-day RSD (%) (n = 18)	5	5.3	3.6
Average conc. (repeatability) (μg/g)	2.87	8.87	30.90
Average conc. (reproducibility) (μg/g)	2.91	9.05	30.28
Absolute recovery (%)*	80	85	86

^*^Absolute recovery expressed as the extraction efficiency when spiked at 4 μg toxin per g brain.

**Table 3:** Subject characteristics.

Disease status	Sample number	SWDBB/ACATU number	Gender	Age Of death	PM Delay	Braak stage	Familial/sporadic	Age onset	Duration of symptoms	Cause of death
Control	3	781	Male	87	24	2	—	—	—	Acute renal failure
Control	13	818	Female	87	47	3	—	—	—	Septicaemia, urinary tract infection, bronchopneumonia
Control	14	355	Male	78	48	1	—	—	—	Uraemic pericarditis, left ventricular hypertrophy
Control	16	826	Female	88	32	2	—	—	—	Caecal carcinoma of colon
Control	17	862	Female	73	50	1	—	—	—	Left ventricular failure, hypertension
Control	18	352	Female	72	24	2	—	—	—	Unknown
Control	19	91	Male	80	67	3	—	—	—	Pulmonary embolism, blood pressure, stomach cancer
Control	21	870	Female	90			—	—	—	Unknown
Control	22	786	Male	85	30.5	2	—	—	—	Acute myocardial infarction due to ischaemic heart disease
Control	24	803	Male	77	42	1	—	—	—	Bronchopneumonia/left cerebrovascular event/atrial fibrillation
Control	25	402	Male	76	23	2	—	—	—	Pneumonia
Control	27	336	Female	82	37	2	—	—	—	Left ventricular failure, renal failure, obstructed bowel
Control	28	259	Male	89	91	2	—	—	—	Bronchopneumonia, chronic obstructive airways disease
Control	29	206	Female	73	59	1	—	—	—	Congestive cardiac failure
Control	30	269	Female	83	24	2	—	—	—	Ventricular failure, ischaemic heart disease
Control	31	20101067*	Female	98	59	3	—	—	—	Unknown
Control	35	20100742*	Male	67	22	0	—	—	—	Unknown
Control	37	20110891*	Male	73	25	0	—	—	—	Unknown
Control	44	20100729*	Male	70	72	0	—	—	—	Unknown
Control	61	851	Female	68	38.75	0	—	—	—	Unknown
AD	1	679	Male	82	110	4	Sporadic	77	5	Dementia
AD	2	702	Female	76	11	5	Sporadic	69	7	Unknown
AD	4	427	Male	75	54	—	Sporadic	66	9	Unknown
AD	5	223	Female	71	67	5	Sporadic	63	8	Serious bacterial infection
AD	6	670	Male	83	85	6	Sporadic	68	15	Unknown
AD	7	531	Female	79	27	6	Sporadic	—	—	Unknown
AD	8	868	Female	88	28	6	Sporadic	85	3	Bronchopneumonia
AD	9	696	Male	78	49	6	Sporadic	65	23	Dementia
AD	10	665	Male	88	75	5	Sporadic	83	5	Bronchopneumonia
AD	11	140	Male	87	71	—	Sporadic	—	—	Unknown
AD	12	697	Female	87	36	—	Sporadic	78	9	Unknown
AD	15	565	Female	75	21	6	Sporadic	69	6	Bronchopneumonia
AD	20	540	Female	92	24	6	Sporadic	87	5	Unknown
AD	23	599	Male	86	72	6	Sporadic	84	2	Unknown
AD	26	833	Female	73	50.5	3	Sporadic	66	7	Ischaemic heart disease
AD	33	20100505*	Female	86	47	6	Sporadic	—	—	Unknown
AD	38	20100725*	Female	87	54	6	Sporadic	—	—	Unknown
AD	39	20100320*	Male	88	84	6	Sporadic	—	—	Unknown
AD	57	839	Male	75	39.5	5	Sporadic	65	10	End stage Alzheimer’s disease
AD	70	424	Female	90	21	4	Sporadic	83	7	Unknown

AD, Alzheimer’s disease.

^*^Newcastle Brain Tissue Resource samples.

**Table 4:** Optimized MRM transitions for DAB, BMAA and D_3_-BMAA, including quantifier ion (Q) and qualifying ion (q).

Compound	Precursorion (m/z)	Cone voltage (V)	Quantifier fragment (Q) (m/z)	Collision energy (eV)	Qualifier fragment (q) (m/z)	Collision energy (eV)
DAB	585.05	10	170.1	45	129	45
BMAA	585.25	10	170.1	45	277.1	25
D_3_-BMAA	588.25	20	170.1	40	—	—
